# Quantifying Shape Transition in Anisotropic Plasmonic Nanoparticles through Geometric Inversion. Application to Gold Bipyramids

**DOI:** 10.1021/acs.jpclett.4c00582

**Published:** 2024-04-02

**Authors:** José Luis Montaño-Priede, Ana Sánchez-Iglesias, Stefano Antonio Mezzasalma, Jordi Sancho-Parramon, Marek Grzelczak

**Affiliations:** † Centro de Física de Materiales (CSIC-UPV/EHU), Paseo Manuel de Lardizabal 5, 20018 Donostia-Sebastián, Spain; ‡ Materials Physics Division, Laboratory of Optics and Optical Thin Films, Rud̵er Bošković Institute, Bijenička cesta 54, 10000 Zagreb, Croatia; ¶ Institute for advanced Neutron and X-ray Science (LINXS), Lund University, IDEON Building: Delta 5 Scheelevägen 19, 223 70 Lund, Sweden; § Centro de Física de Materiales (CSIC-UPV/EHU), and Donostia International Physics Center (DIPC), Paseo Manuel de Lardizabal 5, 20018 Donostia-Sebastián, Spain

## Abstract

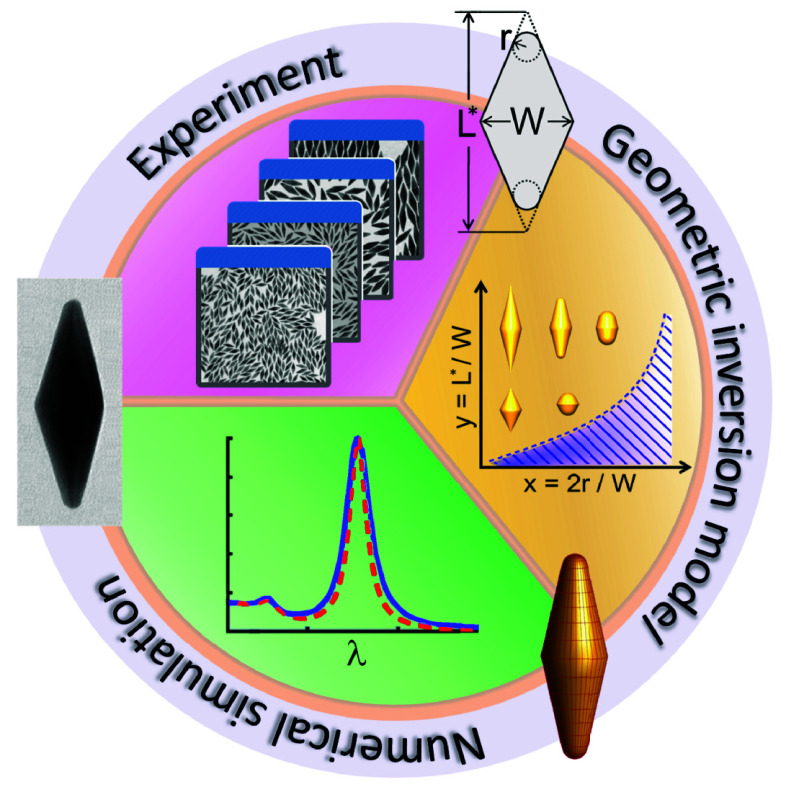

Unraveling the nuanced interplay between the morphology and the optical properties of plasmonic nanoparticles is crucial for targeted applications. Managing the relationship becomes significantly complex when dealing with anisotropic nanoparticles that defy a simple description using parameters like length, width, or aspect ratio. This complexity requires computationally intensive numerical modeling and advanced imaging techniques. To address these challenges, we propose a detailed structural parameter determination of gold nanoparticles using their two-dimensional projections (e.g., micrographs). Employing gold bipyramids (AuBPs) as a model morphology, we can determine their three-dimensional geometry and extract optical features computationally for comparison with the experimental data. To validate our inversion model’s effectiveness, we apply it to derive the structural parameters of AuBPs undergoing shape modification through oxidative etching. In summary, our findings allow for the precise characterization of structural parameters for plasmonic nanoparticles during shape transitions, potentially enhancing the comprehension of nanocrystal growth and optimizing plasmonic material design for various applications.

Anisotropic plasmonic nanoparticles stand out in finely tuning their localized surface plasmon resonance (LSPR) and creating strong electric fields by precisely adjusting the nanoparticle aspect ratio, particularly the length-to-width ratio, surpassing the impact of size variations in isotropic nanoparticles.^1−3^ While gold (Au) nanorods remain the most versatile and consolidated type of anisotropic nanoparticles,^4−7^ Au bipyramids (AuBPs) become an optimal alternative due to a superior number of physical properties and applications across plasmon resonance,^4,8−10^ electric field enhancement,^4,9,11−14^ thermal stability,^15^ coupling processes,^15−22^ sensing capabilities,^23−31^ photocatalysis,^32,33^ acoustic characteristics,^34^ and nonlinear optics.^35−37^ Tuning the shape and size of plasmonic nanoparticles under optical excitation can initiate/elicit chemical transformations near/on their surfaces.^38^ These processes leverage the enhanced localized electric fields associated with plasmon excitation (by photothermal effect and/or hot carriers generation), presenting possibilities to drive reduction or oxidation reactions.^39−43^ Also, AuBPs are particularly appealing for bottom-up fabrication, offering a wide range of structural diversity in two-dimensional (2D) systems^44^ and three-dimensional (3D) supercrystals.^45^ Interestingly, this nanoparticle shape is poorly understood in terms of its relationship between optical properties and shape parameters, as it is due to the high sensitivity of the plasmon band position to sharp edges, such as their tips. The vast number of experimental works are loosely supported by numerical simulations of their optical properties, a fact which is due to the inherent difficulty of accurately matching computed spectra with the experimental response. The intricate nature of these sensitivities presents a substantial obstacle to achieving a comprehensive understanding of AuBPs, emphasizing the need for further research and methodological advancements in simulating their behavior.

For shapes like sphere, cube, and rod, inferring the geometric parameters from electron microscopy micrographs (2D projection of 3D objects) may lead to acceptable predictions of the optical properties.^46^ For bipyramidal shapes, this matter can get very difficult as the curvature radius of highly reactive tips can change upon postsynthetic processing (e.g., thermal treatment, ligand exchange). In a recent investigation, it was demonstrated that employing a truncated bicone model proves to be effective for extracting precise geometric parameters from transmission electron microscopy (TEM) or small-angle X-ray scattering (SAXS) experiments.^47^ Another study showed the spherical tipped bicone to be the best shape for computing LSPR spectra of chemically synthesized AuBPs.^48^ However, arriving at such a conclusion is not as straightforward as it might initially appear. While none of the presumed models can be considered entirely realistic, they ultimately fall into the category of (inverse) ill-posed problems,^49^ dealing with the representation and properties of realistic 3D particles based on idealized geometric projections or restricted information in 2D. Interestingly, if we shift this perspective away from the realm of geometry or topology, the primary objective would be not achieving a perfect correspondence between theoretical and experimental nanoparticle shapes. Paradoxically, it becomes acceptable to deviate from a precise match. To establish a reproducible correction suitable for integration with electromagnetic simulation, one might even consider conjecturing artificial shapes that only numerically fit the experimental data. Delving into an exhaustive discussion about the accuracy of the inverse problem would, therefore, diminish in importance. The key criteria become instead the simplicity of the calculation and the essentiality of the model.

Herein, we propose a geometric inversion model that extends the available experimental parameters (length, width, and area) of 2D projections to the tip radius and cusp-to-cusp length. The full geometric description allows for generating a 3D model as an input for numerical simulations (Figure 1). The accuracy of our model was tested by confronting it with the optical response of experimental AuBPs that gradually undergo chemical etching. In this process, an increase of the tip curvature is accompanied by a decrease of the aspect ratio, leading to pronounced blueshift of the LSPR band. Strikingly, the agreement between numerical and experimental spectra is comparable to those coming from a more sophisticated tool, such as 3D electron tomography.^50−52^ Similar analyses are expected to be profitably applicable to further nanoparticle shapes.

**Figure 1:**
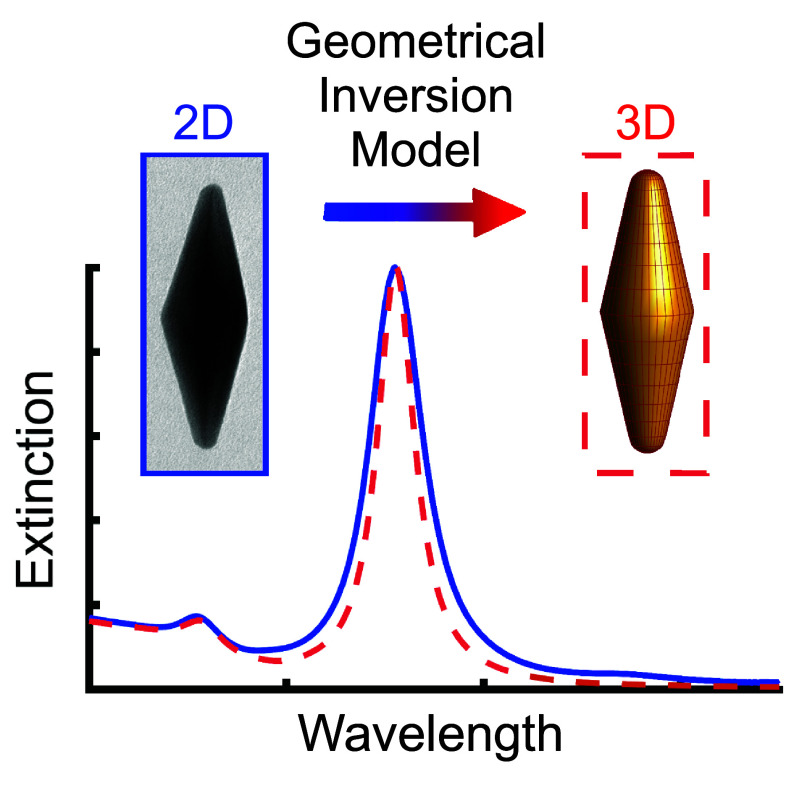
Suggested geometric inversion technique involves transforming a 2D projection (such as a TEM micrograph) of a nanocrystal ensemble into a detailed 3D model. This process aims to assess the reproducibility of experimental optical properties, providing a means to gain more profound insights into the correlation between morphology and optical response.

*The Projective Model*. The choice of a reference model for transitioning from a 2D to a 3D description may take advantage of a number of options of increasing mathematical precision. The Supporting Information (B, second subsection), for example, reports a bipyramid model relying on three shape descriptors, two of which rebuild the geometry by means of distinct curvature radii at both the tips and lateral sides. This approach, in spite of supposing equal curvatures at opposite angles (i.e., an oversimplification), is already quite tough to be confronted with experimental data and be dealt with computationally. As mentioned above, respecting every 3D constraint, dictated by opportunely fixing bipyramid length, width, and shape descriptors, is not an easy task, and a suspicion arises that it may even be unnecessary for predicting an optical response with good accuracy.^48^ Preliminary numerical extinction spectra (Figure S4) further reinforce the utilization of the bicone model. These calculations exhibit a closer alignment with the experimental spectrum compared to considering a pentagonal base. These findings robustly support this choice, affirming its effectiveness in accurately representing the macroscopic response, simplifying the calculations, in order to reproduce experimental observations despite minor geometric deviations. For this reason, we preferred to switch to a two-descriptor model, employing as a reference shape a bicone with rounded tips, whose projection in two dimensions is illustrated in Figure 2a. Tips are defined by sectors of a circle, delimited by orthogonal radii to the conic projections. While it is still a simplification, this choice posits a clear definition for the curvature radius at both the top and the bottom of nanoparticles (*r*).

**Figure 2:**
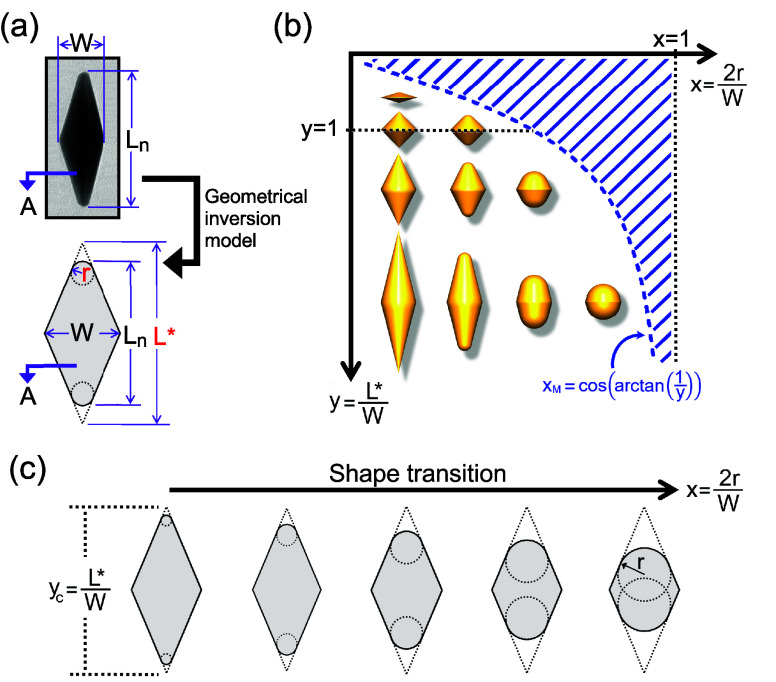
(a) Schematic representation of the proposed geometric inversion of the 2-descriptor model for rounded bipyramids. By applying it to the parameters extracted from a real bipyramid projection (TEM micrographs) [width (*W*), net length (*L*_n_), and projected area (*A*)], we determine the cusp-to-cusp length (L*) and tip curvature radius (*r*), facilitating the generation of a corresponding 3D particle model for the actual bipyramid. (b) A type of geometric phase diagram for the presented inversion, with rescaled descriptors *x* = 2*r*/*W* and *y* = L*/*W*. An increase in *x* accentuates the tip curvature, spanning from a bipyramid with sharp tips (*x* = 0) to a perfect sphere (*x* = 1, *y* → *∞*). The blue dashed line defines the valid region of the model for *x* < *x*_M_; beyond this, there is no solution. As *y* denotes the cusp-to-cusp aspect ratio, reaching *y* = 1 describes a bipyramid with equal width and cusp-to-cusp length, establishing the boundary between elongated (*y* > 1) and compressed (*y* < 1) bipyramid models. This research focuses on bipyramids in the region where *y* > 1, while those with *y* ≤ 1 exhibit nanoparticle models spanning from unitary cusp-to-cusp aspect ratios to plate-like topology. (c) Shape transitions such as in the oxidative etching process, where the cusp-to-cusp aspect ratio is maintained at a constant value (i.e., *y*_c_) and the rescaled radius (*x*) increases, results in a reduction of both net length and area.

There are two longitudinal characteristic sizes in this construction: the cusp-to-cusp length of the bicone (L*) and that of the real particle, devoid of tip curvatures (*L*). While *L* = *L*_n_ – 2*r* is built on the net length of the real particle (*L*_n_), the former is model-dependent, as it comes from an extrapolation conducted in realistic samples. The only possibility to detect L* would be producing nanoparticles with perfectly sharp tips (*r* = 0), a situation that is unattainable in practice. On the other hand, were one assuming the real nanoparticle length *L*_n_ ≈ L*, model errors would be definitely high. To simplify the scheme (Figure 2a), we rescaled each geometric feature by *W*, the projected width of the conical base diameter:
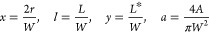
1*a* stands for the rescaled total area of the projected figure, which displays the original value *A* (see Figure 2a), and *y* introduces the cusp-to-cusp aspect ratio, i.e., built on the distance between the two vertices of the circumscribing bicone. From elementary trigonometric arguments, one has (*y* – )^2^ = *x*^2^(1 + *y*^2^) and
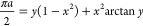
2or
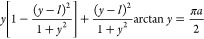
3The last is the basic model equation and is well posed. This can be ascertained from inspecting the limiting behaviors, which take on the expected values
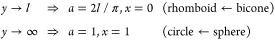
4The case given by *y* → 0 returns no solution. Equation 3 is required to be coupled with statistical data descending from the geometric analysis of nanoparticle samples (Supporting Information, Experimental Section). In our case, however, where *A*, *W*, and *L*_n_ are available, eq 3 is not yet ready to be applied. To this aim, it suffices to rescale *L*_n_ into the actual nanoparticle aspect ratio, _n_ = *L*_n_/*W*, and observe that  + *x* =  leading to
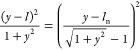
5Now, the system formed by eq 2, eq 3, and eq 5 is now able to invert consistently the 2D figure into a 3D rice grain-like nanoparticle mimicking a bipyramid with zero curvature radii on the lateral sides (i.e., with lateral cusps). In fact, from the experimental knowledge of *a* and _n_, one first deduces *y*, then  from eq 5 and, finally, *x* = _n_ – . The physical value of curvatures at the tip, which is the most relevant unknown, completing the statistical inference, is finally determined from *r* = *Wx*/2. We also note that the difference between the cusp-to-cusp and nanoparticle aspect ratios increases with increasing *x* and *y*. They are nearing each other when , as it is documented in the last paragraph of Supporting Information, section B. For the experimental AuBP samples analyzed here, it turns out *y* – _*n*_ ≈ (1–3) nm.

At a fixed *y*, the nanoparticle projection undergoes a bound given by , where  = arctan *y*^–1^ is half of the angle at the tip. This limit, which at a fixed width implies a maximum radius, *r*_M_, comes from the orthogonality of the tip sector to the conical body and turns out to be a locus point demarcating the allowed nanoparticle domain. The sort of geometric phase diagram in Figure 2b would obviously extend to the entire (*x*, *y*) plane if α were allowed to vary freely. In such a case, however, the model would take on a remarkably higher complexity, probably without adding much to its actual predictability. During the oxidative etching of AuBPs (*vide infra*), we can effectively map the nanoparticle transformation process onto this diagram by maintaining a fixed cusp-to-cusp aspect ratio at a given value, say *y* = *y*_c_, while allowing the rescaled radius *x* to increase (Figure 2c).

Finally, the volume (rescaled as *v* = *V*/*W*^3^) derivation stems from a simple interpretation of the bipyramid as a rotational solid along the longitudinal axis integral to the length L*. The Supporting Information (B, first subsection) reports all the calculation details, presenting the following formula:
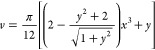
6We may note that the rotational symmetry of the nanoparticle along *y* implies *V* as being fully homogeneous in *W*, partly homogeneous in *r*, and inhomogeneous in L*. Fixing *x* and *y* returns an infinite family of similar nanoparticles, homogeneous in *W*, with equal cusp-to-cusp aspect ratios and rescaled tip radius. To pass from nanoparticle geometry to optical response, one needs to include the bipyramid width and calculate the volume *V*. Note, finally, that eq 6 turns out not to affect the model validity, still set by the function *x*_*M*_=*x*_*M*_(*y*) that was formerly introduced.

Now that this essential projective model and a few geometric data were proven to suffice for obtaining the radius of curvature, one can move forward by verifying if the optical computations are consistent with the experimentally detected plasmonic spectra.

*Experimental Assessment of the Projective Model*. To experimentally assess the geometric inversion model, we synthesized a set of four AuBPs samples^53^ of diverse lengths and volumes and subjected them to TEM analysis to obtain 2D projections (Figure S1). The image analysis (Supporting Information, secion A) allowed obtaining three structural parameters (net length, width, and projected area) that were used to compute the nanoparticle aspect ratio straight away and then, from the knowledge of *x* and *y* (see the projective model), the nanoparticle volume (Table 1).

**Table 1:** Mean and Standard Deviation of the Net Length, Width, and Projected Area from TEM Analysis, and Average Nanoparticle Aspect Ratio^a^ and Volume^b^ (*V*) of As-Synthesized AuBP Samples

Sample	Net Length, *L*_n_ (nm)	Width, *W* (nm)	Area, *A* (nm^2^)	a (−)	Volume,^b^ V (nm^3^)
1	121 ± 4	35 ± 2	2616 ± 132	3.5	49987
2	93 ± 5	30 ± 2	1843 ± 160	3.1	31570
3	71 ± 4	18 ± 1	818 ± 71	3.9	8291
4	49 ± 2	16 ± 1	504 ± 37	3.1	4521

a .

b Determined from eq 6, *V* = *W*^3^*v*.

The four initial samples of AuBPs were subjected to stepwise oxidative etching,^54−56^ giving rise to four series (Supporting Information, section A). The oxidation of Au metal atoms is well documented to occur preferentially at the particle tips, due to their higher curvature (∼1/*r*).^55^ In the present case, oxidizing the tip leads to a transition from a bicone to a rice grain-like shape. TEM analysis of the samples at each oxidative step confirmed (1) a progressive shape transition (Figure 3a; i–vi, sample 1) accompanied by blue-shifting the LSPR band from ≈867 to 620 nm (Figure 3f); (2) a reduction of the nanoparticle net length from *L*_n_ ≈ 121 to 71 nm (Figure 3b); (3) the invariance of the AuBP width, *W* = (35 ± 2) nm (Figure 3c); and (4) a decrease of the projected area, from *A* ≈ 2616 to 1904 nm^2^ (Figure 3d). The observed trend of changing structural parameters upon oxidative etching was ascertained in the other samples (Figures S5 and S7 in Supporting Information, section C).

**Figure 3:**
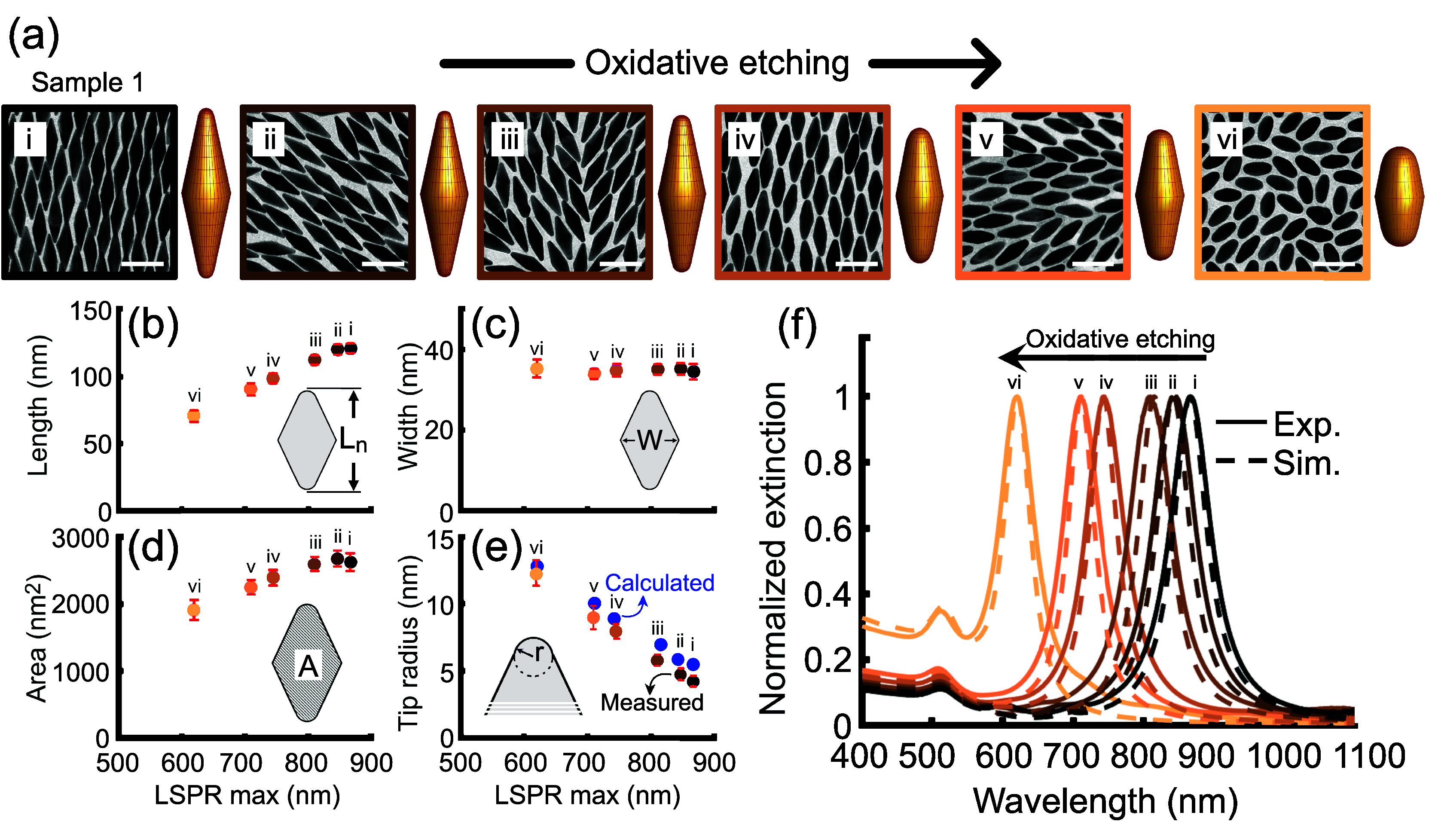
Validation of the proposed geometric inversion model through experimental/computational comparison for an oxidative etching series of the AuBP sample 1. (a) TEM micrographs (scale bars = 100 nm) and corresponding 3D particle models obtained by geometric inversion. Increasing oxidation degrees are indicated by roman numerals from *i* to *vi* (TEM border colors shift from black to light copper), where *i* denotes sample 1 prior to oxidative etching. The arrow above the TEM micrographs points out the direction of the oxidation path in the series. (b–d) Error bar charts show the variability in the measured (b) net length, (c) width, and (d) projected area of nanoparticles derived from TEM micrographs by using ImageJ Fiji software, plotted against the experimental longitudinal LSPR wavelength (error bars are in red). (e) Error bar chart depicting the experimentally measured tip radius which was manually obtained from plotting TEM micrographs against the longitudinal LSPR wavelength. Blue circles in panel e denote the calculated tip radius determined from the geometric inversion model applied to the average values of length, width, and projected area in panels b–d, respectively. (f) Experimental extinction spectra of the series of sample 1 (solid lines) along with simulated extinction cross-sectional spectra of the 3D-model bipyramids. The arrow above the spectra stands again for the path direction of the oxidative etching grade.

Typically, assessing the structural parameters of plasmonic nanoparticles involves matching ensemble experimental extinction spectra with numerically generated counterparts for individual nanoparticles by using an idealized model. To evaluate qualitatively such a match, we calculated numerical extinction cross section spectra (Figure 3f) with the boundary element method^57,58^ (Supporting Information, section C). As an input, we employed the generated 3D objects of nanoparticles at each oxidation stage that were obtained by projective model (Figure 3a). Note that, to generate 3D objects, one needs to calculate the values of L* and *r* by eqs 3 and 5. We found that the agreement between experiments and computations is very good, reflecting the presence of the transverse LSPR and higher-order modes. It extends beyond merely aligning peak positions, which is typically the adopted approach in spectrum matching. There is no necessity to artificially broaden the numerical spectra to match the full width at half-maximum (fwhm) of the experimental response. This underscores the robustness and accuracy of the employed method at our nanolength scales and the high quality of experimental samples. Importantly, the projective model allows for estimation of the tip radius, representing the structural parameter with the largest impact on the final extinction spectra. The tip radii calculated by our inversion method showed a relatively good match with the values evaluated manually from TEM images for each sample (Figure 3e). A slight variance may be ascribed to measurement errors and their statistical propagation, which could result in a larger deviation in the simulated optical properties. In summary, these findings suggest that the projection model enables the swift creation of 3D objects from 2D projections of monocrystals, facilitating the generation of optical data for subsequent comparison with experiment.

The shape transition of the four initial experimental samples through oxidative etching generated a set of 22 samples in total, grouped into four series (Table S2 and Figure S8 in Supporting Information, section C). The volumes across all generated shapes between bicone and sphere covered 2 orders of magnitude, from 4000 to 50000 nm^3^. Setting the volume as an experimental variable is a reasonable means to evaluate the accuracy of the projective model, as the volume is the main parameter affecting the contributions of absorption and scattering cross sections. We conducted a comparative analysis of extinction spectra and corresponding structural parameters for all samples (Figure 4). It was noted that the positions of the maxima of LSPR for experimental and numerical spectra, distributed over 400 nm, coincide relatively well, as evidenced by the high coefficient of determination, *R*^2^ ≥ 0.97 (Figure 4a). However, the metric based on a single data point (LSPR maximum) is insufficient as it does not account for the spectrum width and intensity ratio of longitudinal and transverse plasmon bands. Therefore, we conducted a root-mean-square deviation (RMSD) analysis, encompassing the entire spectrum of *N* = 701 data points spanning from 400 to 1100 nm, as it follows from RMSD ≡ . Here, the summation extends over the *i* = 1, 2, ... *N* wavelength samples within the spectral range, and  represents the squared difference between the experimental and simulated normalized intensities at the *i*-th wavelength. The lower the RMSD value, the better the match between the experimental and numerical extinction spectra. Figure 4b displays the RMSD versus particle volume (eq 6, *V* = *W*^3^*v*) of our four series. Series 1, which exhibits the largest volume, shows low RMSD (∼0.05) for the entire shape transformation; that is, all data points are more clustered in this representation. As the particle volume decreases, the RMSD extends across a broader range, peaking at 0.15 for particles with the smallest volume (Figure 4b). We postulate that the RMSD discrepancy in small-volume nanoparticles accounts for inherent limitations of electrodynamics simulations. Classical electrodynamics faces challenges in accurately capturing the electric field response generated by charges confined to small regions, i.e., the biconical tips.^59^ This reasoning is supported by decreasing the RMSD for nanoparticles undergoing oxidative etching, as it follows from the increase of the tip radius (Figure 4b). A potential avenue for addressing this challenge is incorporating in the simulations size and shape corrections to the refractive index of the nanoparticle material (finite-size or quantum-like effects).^59^ Delving into this aspect, however, would fall outside our aims, which are mainly focused on validating the geometric inversion method.

**Figure 4:**
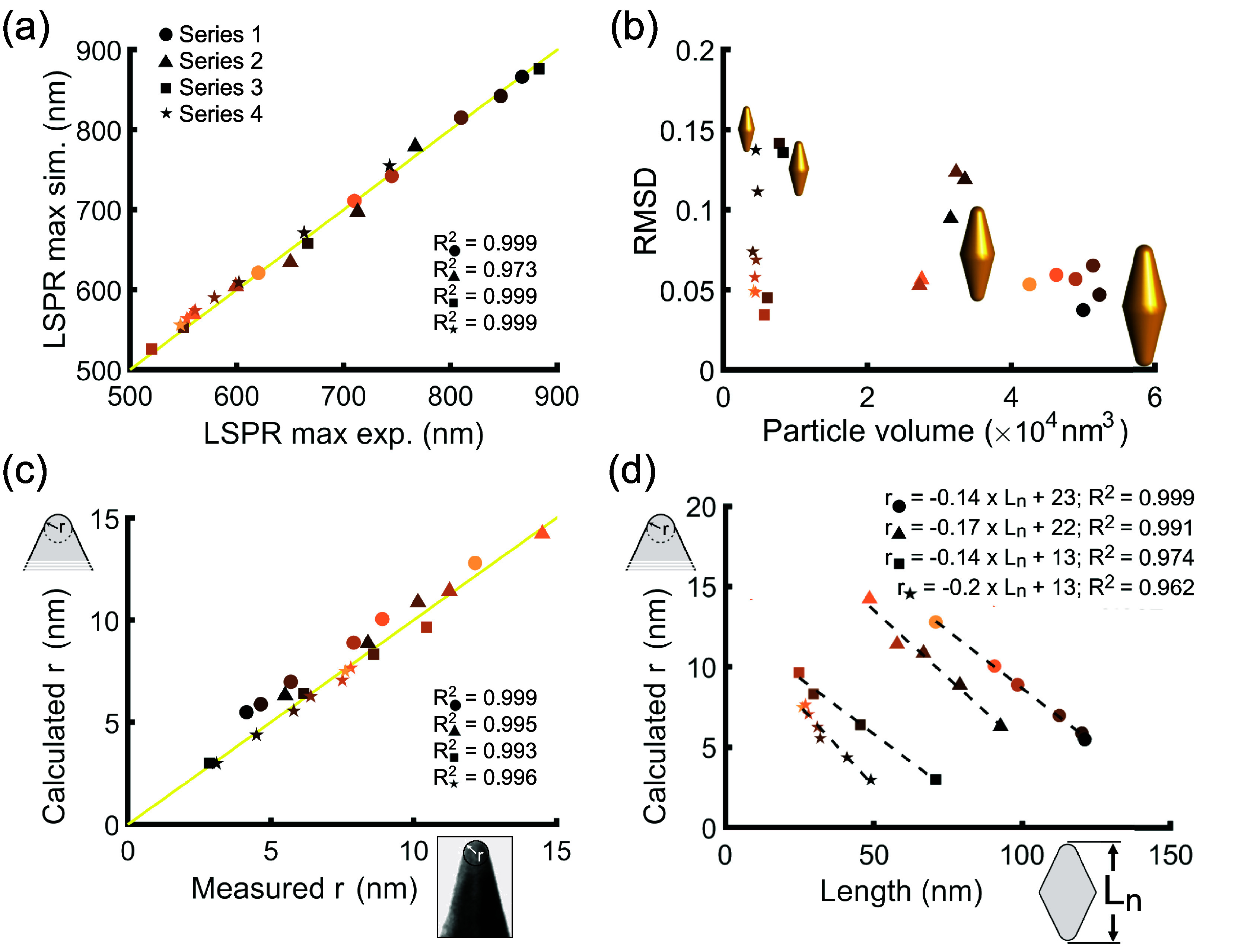
Comparative analysis of simulated and experimental optical properties across all etched (oxidized) series of samples 1–4. (a) Comparison between experimental and simulated spectral positions of the LSPR maximum. (b) Root-mean-square deviation (RMSD) of simulated extinction spectra relative to experimental results against particle volume (eq 6, *V* = *vW*^3^). (c) Comparison between measured (TEM analysis) and calculated tip radii (geometric inversion model). (d) Correlation between net length and calculated tip radius, revealing a linear relationship (dashed black lines) in each series, suggesting exclusive tip-oxidation of AuBPs. From our model, the best fit coefficients in *r* = *AL*_*n*_ + *B* depend on L* and *W* by 1/*A* = −L*/*B* = 2[1 – ] < 0. Replacing the obtained values, which are displayed in the top right inset, we derive L* = 164.3, 129.4, 92.9, 65.0 nm, along with *W* = 36.8, 34.0, 20.8, 19.4 nm for samples 1–4, respectively.

Given that the alteration of the tip radius predominantly affects the LSPR position, we examined the correlation between the tip radii estimated manually from TEM micrographs and those computed by the geometric inversion model (Figure 4c). The correlation plot between measurements and calculations displays a negligible deviation (*R*^2^ ≥ 0.993). Worth mentioning is the fact that measurement errors in determining the tip radius from TEM micrographs result in a larger disparity between calculated and experimental extinction spectra compared to those coming from geometric inversion, as witnessed by the corresponding RMSD plot (Figure S9).

The progressive oxidative etching of AuBPs for each series evidenced a nearly linear relationship between the tip radius and nanoparticle length (Figure 4d). With increasing tip radius, the nanoparticle length decreases linearly, a trend that is independent of the initial particle length. This is consistent with the projective model at a constant *y*, implying . By this equation, we can infer L* and *W* in each series from the best fits in Figure 4d. The obtained values are reported in the caption, exhibiting satisfactory concordance with model predictions. From the physical point of view, this behavior means that oxidative etching takes place exclusively at the AuBP tips. With each oxidation step, the reactive surface area at the nanoparticle ends increases, making more space available for the next oxidation step.^60−63^ Note that the high coefficient of determination (*R*^2^ ≥ 0.962 value) suggests that the etching process was conducted with high precision. Undoubtedly, enhancing the reactive surface area should boost the oxidation kinetics at each stage given that the quantity of Au atoms at the tip is the rate-determining step. We are aware that conducting a kinetic study falls outside the scope of the present work. However, our geometric inversion model is also promising to offer novel tools to describe quantitatively the processes taking place on the nanoparticle surface. It opens avenues for additional implementation to monitor the real-time shape transition, aiding in comprehending the mechanisms underlying nanocrystal growth.

*Beyond the Experimental Parameter Space*. The selected dimensions of initial AuBPs (Table 1) were imposed by the limitations of the existing experimental protocols.^53^ The derived shape descriptors, however, go beyond such limitations and open up a new geometrical landscape (Figure 2b) that can be explored to find optimal morphological parameters that match specific optical properties and, therefore, targeted applications. For example, in colorimetric sensing involving oxidative etching, it becomes crucial to identify the dimensions yielding the most significant change in the LSPR position within the extinction spectrum in the visible range.^26,36,64^ On the other hand, when designing thermoplasmonic nanoparticles^65^ for applications such as hyperthermia treatment, the focus shifts toward determining the dimensions where absorption gets maximized at the specific wavelength of interest.^15,18^ More generally, studying the thermoplasmonic response concerning the geometry and topology of nanoparticles enables the prediction of local temperature gradients and the control of thermal effects in the surrounding medium when particles are illuminated. This is especially relevant in scenarios with energy-dependent photocatalytic phenomena, such as the various regimes in (chiral) crystal nucleation and growth.^66^ Conversely, for applications in biological detection and diagnosis, with an emphasis on reducing losses, it is imperative to precisely identify the sizes that maximize scattering with minimal absorption and align with the spectral window of tissues.^28−31^ Each potential usage demands a tailored approach in the exploration of the most effective plasmonic bipyramid configuration. A primary implication of the proposed geometric inversion model lies in its ability to predict optical properties, enabling a streamlined approach to prototyping the optimal configuration for a specific interest. As an illustrative example, Figure 5 showcases the maximum theoretical extinction, absorption, and scattering cross sections across a broad range of the former shape descriptors (*x*, *y*) for *W* = 35 nm. These results are visualized in maps, reporting the magnitude (upper panels) and spectral position (lower panels) of the maximum light extinction, absorption, and scattering cross sections of the bipyramids. As a reference, we have plotted points on the maps corresponding to the oxidative-etching series of sample 1 (Figure 3). These representations reveal a wide range of possible nanoparticle dimensions, providing a valuable geometrical landscape for further exploration to meet given applications of interest.

**Figure 5:**
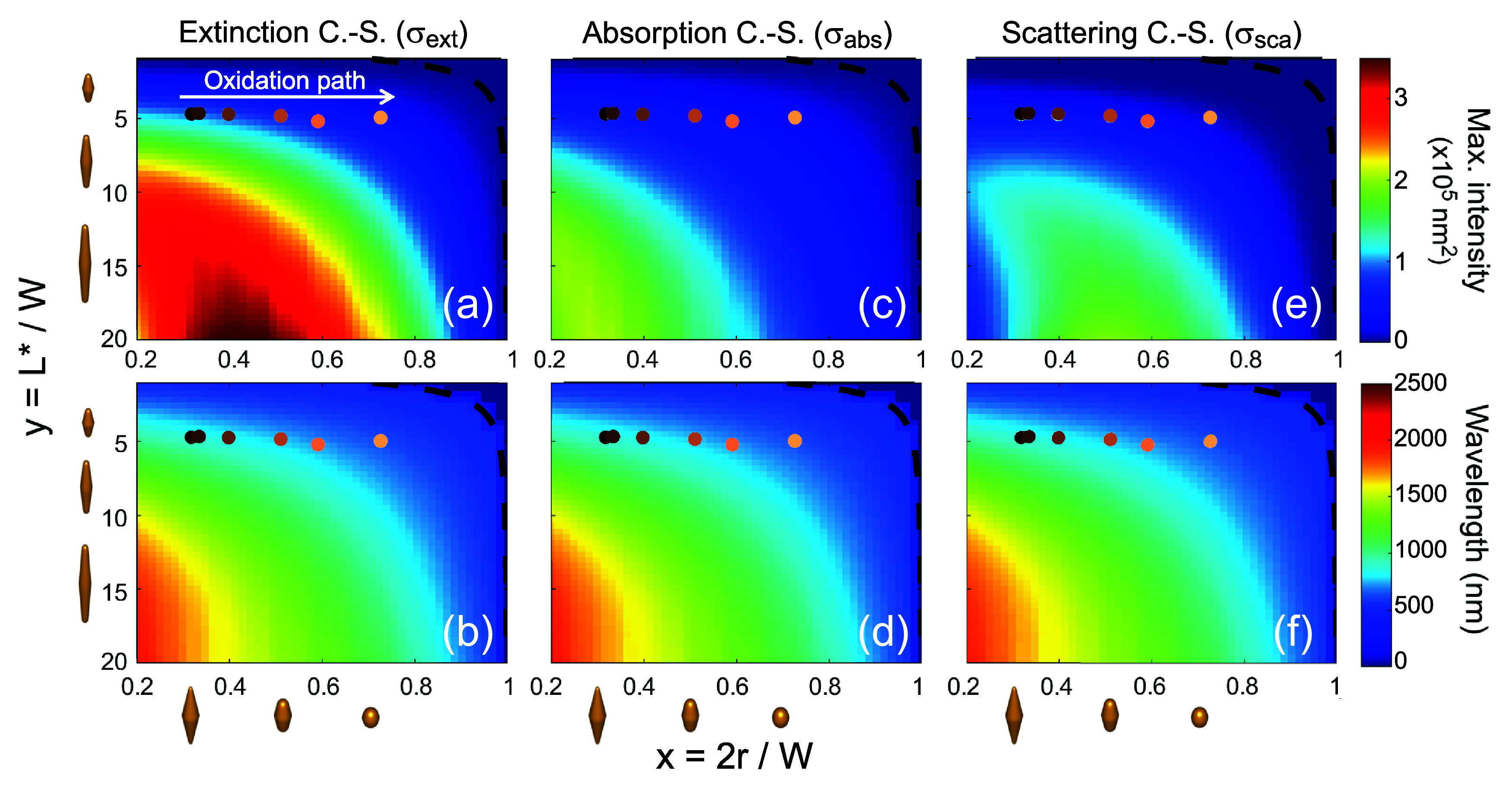
Magnitude (upper panels) and wavelength position (lower panels) of (a and b) extinction, (c and d) absorption, and (e and f) scattering cross section maxima, covering the bipyramid shape descriptor range 0.2 ≤ *x* ≤ 1 (abscissa) and 1 ≤ *y* ≤ 20 (ordinate) for *W* = 35 nm. The 3D particle models reported below the abscissas and next to the ordinates serve as visual references, illustrating the shape change along the two axes. Shape parameters of the etched (oxidized) series of sample 1 are represented by circle markers with the same reference colors as in Figure 3, where the white arrow in panel a points in the direction of the oxidation path. Black dashed lines in all the panels represent the bound (model validity region) *x*_M_ = cos(arctan *y*^–1^) as in Figure 2.

While the wavelength of the peak extinction varies monotonically with both *x* and *y* (see Figure 5b,d,f), the magnitude behavior is more intricate (see Figure 5a,c,e). When *y* is within the range of approximately *y* < 15, the gradual rounding of the bipyramid tips due to particle oxidation (indicated by the white arrow in Figure 5a) leads to a decrease in the maximum light extinction and a simultaneous blue-shift. However, for *y* > 15, the light extinction initially increases, followed by a subsequent decrease as the tips undergo rounding. This trend is evident in the absorption and scattering intensities as well (see Figure 5c–e). In each case, there exists a region where the cross-sectional intensities, at a fixed *y*, reach a maximum as a function of *x*, the tip radius (see Figure 5a,c,e). These regions do not coincide, but the peak in light extinction occurs when both absorption and scattering tend to have high values. This phenomenon is particularly pronounced in intermediate-oxidized bipyramids with high cusp-to-cusp aspect ratios. Specifically, within the studied region, three main stages are observed during the oxidation of bipyramids with large cusp-to-cusp aspect ratios: (1) moderately high absorption and low scattering (very sharp tips); (2) high absorption and high scattering (moderately rounded tips); (3) high scattering and low absorption (very rounded tips).

In summary, our study of gold bipyramids, supported by a novel geometric inversion model, reveals a dynamic interplay between shape parameters and optical properties during oxidative etching. The model accurately predicts key structural parameter tip radius, enabling precise simulations validated against experimental spectra. Our model enables the generation of numerical spectra that closely align with experimental responses on a macroscopic scale that was quantitatively assessed by root-mean-square deviation across the entire spectral range, providing a simpler and faster workflow for determining the quality of gold bipyramids. Despite challenges in electrodynamics simulations for small nanoparticles, our approach demonstrates reliability. Of course, we foresee future benchmarking of our method using other means, such as electron tomography or scattering techniques. Progressive oxidative etching reveals a negative linear regression between tip radius and nanoparticle length, offering new directions in studying the growth mechanism of anisotropic gold nanocrystals. The shape descriptors for gold bipyramids enabled modeling a large optical parameter space that can serve as a roadmap for further tailoring of these structures for targeted applications in sensing and plasmonic catalysis. Overall, this research strengthens our understanding of the relationship between shape and optical properties in plasmonic nanoparticles and establishes the proposed inversion model as a robust tool for nanophotonics and the strategic design of any plasmonic structure with various applications.

## Supporting Information Available

A. Experimental Part: materials, synthesis of gold bipyramids, partial etching of gold bipyramids, and instrumentation; B. Modeling: volume calculation in the geometric inversion model, three-descriptor analysis of bipyramid shapes, and model aspect ratios; C. Simulation of Optical Properties (PDF)
