# Supplementary material for: Quantifying Shape Transition in Anisotropic Plasmonic Nanoparticles through Geometric Inversion. Application to Gold Bipyramids
Source: J Phys Chem Lett. 2024 Apr 2;15(14):3914–22. doi: 10.1021/acs.jpclett.4c00582 (PMC11017706; doi:10.1021/acs.jpclett.4c00582)
Supplement: Supplementary file 1 — jz4c00582_si_001.pdf [file jz4c00582_si_001.pdf]

Quantifying Shape Transition in Anisotropic Plasmonic Nanoparticles through Geometric Inversion: Application to Gold Bipyramids - Supporting Information

José Luis Montaño-Priede,[†] Ana Sánchez-Iglesias,[†] Stefano Antonio Mezzasalma,[*,‡,¶] Jordi Sancho-Parramon,[*,‡] and Marek Grzelczak[*,§]

†*Centro de Física de Materiales (CSIC-UPV/EHU), Paseo Manuel de Lardizabal 5, 20018 Donostia-Sebastián, Spain*

‡*Materials Physics Division, Laboratory of Optics and Optical Thin Films, Ruđer Bošković Institute, Bijenička cesta 54, 10000 Zagreb, Croatia*

¶*Institute for advanced Neutron and X-ray Science (LINXS), Lund University, IDEON Building: Delta 5, Scheelevägen 19, 223 70 Lund, Sweden*

§*Centro de Física de Materiales (CSIC-UPV/EHU), and Donostia International Physics Center (DIPC), Paseo Manuel de Lardizabal 5, 20018 Donostia-Sebastián, Spain*

E-mail: stefano.mezzasalma@irb.hr; jsancho@irb.hr; marek.g@csic.es

1 A. Experimental Part

1.1 Materials

Gold (III) chloride trihydrate ($HAuCl_4$, $\geq$ 99.9 %), hexadecyltrimethylammonium chloride (CTAC, 25 % in water), citric acid (99 %), sodium borohydride ($NaBH_4$, 99 %), hexadecyltrimethylammonium bromide (CTAB, $\geq$ 99.0 %), ascorbic acid (AA, $\geq$ 99%) and silver nitrate ($AgNO_3$, $\geq$ 99%) were purchased from Aldrich-Merck. Hydrochloric acid (HCl, 37 %) was purchased from Scharlau. All chemicals were used without further purification. Milli-Q water (resistivity 18.2 MΩ·cm at 25 °C) was used in all experiments. All glassware and stirrer bars were washed with aqua regia, rinsed with Milli-Q water and dried before use.

1.2 Synthesis of Gold Bipyramids

Gold bipyramids (AuBPs) were synthesized according to a previously reported procedure using seed-mediated growth.[1] The gold seeds were prepared by fast reduction of gold precursor with $NaBH_4$ in the presence of both a cationic surfactant (CTAC) and citric acid in a 20 mL scintillation vial. To an aqueous mixture containing CTAC (10 mL, 50 mM), $HAuCl_4$ (0.05 mL, 50 mM), and citric acid (0.05 mL, 1 M), freshly prepared $NaBH_4$ (0.25 mL, 25 mM) was added under vigorous stirring at 20 °C. The solution turned from yellow to brownish immediately. Two minutes later, the seed solution was heated at 80 °C for 90 min in a silicone oil bath, under gentle stirring. Its color turned gradually from brown to red, indicating an increase in nanoparticle size. Finally, the solution was removed from the oil bath, stored at room temperature, and used without further treatment. Gold bipyramids with different dimensions were prepared by adding a certain volume of gold seeds (0.9, 1.1, 2.0, and 3.7 mL, for the preparation of the samples 1, 2, 3 and 4, respectively) under vigorous stirring to a growth solution comprising CTAB (100 mL, 100 mM), $HAuCl_4$ (5 mL, 10 mM), $AgNO_3$ (1 mL, 10 mM), HCl (2 mL, 1M) and AA (0.8 mL, 100 mM), at 30 °C. Five minutes later, the mixture was left undisturbed at 30 °C for 2 h. AuBPs were washed by centrifugation (2 cy-

cles at 7000 rpm, 30 min), to remove excess reagents. After the second centrifugation cycle, nanoparticles were redispersed in CTAB (100 mM) to a final gold concentration of 0.5 mM. The resulting dimensions of AuBPs are presented in Table 1 of the main text. Absorbance spectra and TEM micrographs of the as-synthesized AuBPs are shown in Figure S1.

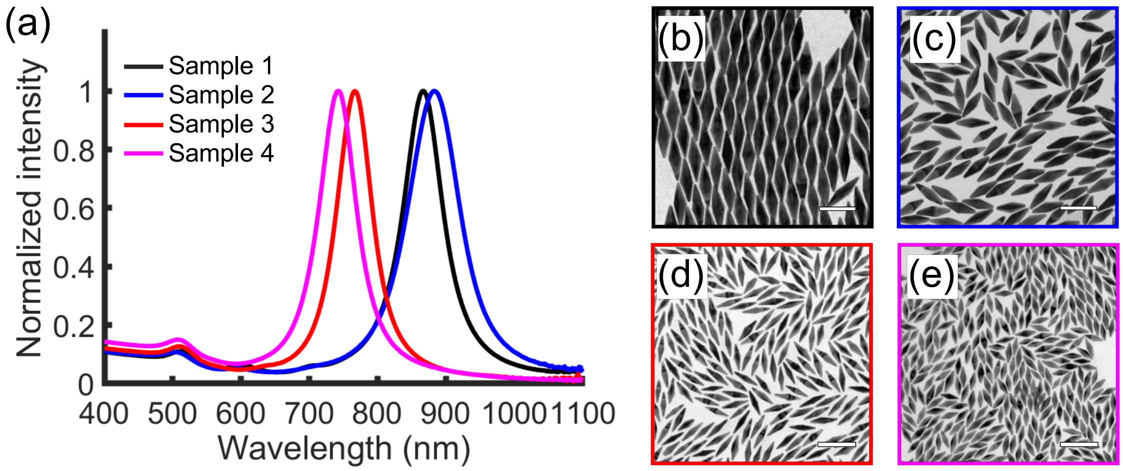

Figure S1: a) UV-Vis-NIR absorbance spectra of the synthesized bipyramids prior to oxidative etching process. TEM micrographs of initial samples. Scale bars = 100 nm.

1.3 Partial Etching of Gold Bipyramids

Oxidative etching of AuBPs was performed by means of the Au^{+3}-CTAB complex.[2,3] Different samples of etched bipyramids were prepared by using nanoparticles obtained as described above. To a solution containing AuBPs (10 mL, 0.5 mM) in CTAB (100 mM), an aliquot of Au^{+3}-CTAB complex ([$HAuCl_4$] = 1 mM, [CTAB] = 100 mM) was added dropwise at 30 °C under vigorous stirring for 20 min (Table S1). Subsequently, the solutions were centrifuged twice (9000 rpm, 30 min) and redispersed in water.

1.4 Instrumentation

Optical extinction spectra were recorded with an Agilent Cary 3500 UV-visible spectrophotometer. TEM images were acquired with a FEI Tecnai G2 20 TWIN transmission electron microscope (TEM) operating at an acceleration voltage of 200 kV. The dimensions of AuBPs

Table S1: Volume of Au^{+3}-CTAB complex used for oxidative etching of different AuBP samples, while keeping constant metallic Au concentration in all the experiments (10 mL, $[Au^0] = 0.5$ mM).

	Volume of Au^{+3}-CTAB complex / μL					
Sample	ii	iii	iv	v	vi	vii
1	100	200	300	400	500	-
2	100	300	500	600	-	-
3	188	219	250	-	-	-
4	110	150	200	233	250	270

were determined by TEM image analysis, measuring over 200 randomly selected nanoparticles.

2 B. Modelling

2.1 Volume Calculation in the Geometric Inversion Model

For deriving the biconical volume (V), we interpret the nanoparticle as a rotational solid. Setting the origin to the center of mass and introducing a reference system with abscissa χ, directed along L^*, and with ordinate axis along W, it turns out:

$$V \;=\; 2\pi \int_{\mathcal{D}_c} f_c^2(\chi) d\chi \;+\; 2\pi \int_{\mathcal{D}_r} f_r^2(\chi) d\chi \tag{S1}$$

where $f_c = f_c(\chi)$ and $f_r = f_r(\chi)$ are the functions describing respectively the truncated cone and the curved sector in the first quadrant ($\chi \geq 0$, $f_{c,r} \geq 0$). It is convenient to operate in the same non-dimensional units parametrizing the model projection, thus we define $X = \chi/W$ to obtain:

$$f_c(X) \;=\; \tfrac{1}{2} - \tfrac{1}{y} X \tag{S2}$$

$$f_r(X) \;=\; \tfrac{1}{2}\sqrt{x^2 - (2X - \ell)^2} \tag{S3}$$

where x, y, ℓ are defined as in the main text. A simple inspection of the integration limits shows that $\mathcal{D}_c = [0, \frac{\ell}{2} + \frac{x}{2\sqrt{1+y^2}})$, $\mathcal{D}_r = [\frac{\ell}{2} + \frac{x}{2\sqrt{1+y^2}}, \frac{\ell+x}{2})$.

Replacing the former two equations in the volume expression returns in the first instance:

$$\begin{aligned} v \;=\; \frac{\pi}{12y^2} \Bigg\{ & \frac{x^3}{\sqrt{(1+y^2)^3}} \left[1 - y^2(2 + 3y^2)\right] \;+\; \frac{3x^2}{1+y^2}(\ell - x) \;+ \\ & + \frac{3x}{\sqrt{1+y^2}}(\ell - x)^2 \;+\; \ell^2(\ell - 3y) \;+\; y^2(2x^3 + 3\ell) \Bigg\} \end{aligned} \tag{S4}$$

with $v = V/W^3$. To get the final result, as reported in the main text, it does suffice recalling the constraint $\ell - y = -x\sqrt{1+y^2}$ and one is left with a sum of three terms:

$$v(x, y) \;=\; \frac{\pi x^3}{6} \;+\; \frac{\pi y}{12} \;-\; \frac{\pi x^3}{12} \frac{2 + y^2}{\sqrt{1+y^2}} \tag{S5}$$

This function can recover upon the equivalent limit conditions a pure biconical volume, V_c, with height $h = L^*/2$ and base radius $R = W/2$:

$$V_c \;=\; W^3 \lim_{x\,\to\,0^+} v(x,y) \;=\; \tfrac{\pi}{12}(2h)(2R)^2 = \; 2\cdot\tfrac{\pi}{3}hR^2 \tag{S6}$$

and a pure spherical volume, V_r:

$$V_r \;=\; W^3 \lim_{\substack{x\,\to\,1^-\\ y\,\to\,\infty}} v(x,y) \;=\; \tfrac{\pi}{6}(2R)^3 \;=\; \tfrac{4\pi}{3}R^3 \tag{S7}$$

where it is implicitly meant $y/x^3 - y^4/\sqrt{(1+y^2)^3} \to 0^+$.

The biconical limit corresponds to the ordinate axis in Figure 2 of the main text, while the spherical one is recovered by the point at infinity of the frontier demarcating the validity region of the projective model ($x \le x_{_M}(y) = \cos\arctan 1/y$, blue line). Observe that the expressions for V_c and V_r would equally stem from Eq. (S4) upon the same limits, with the additional conditions $\ell \to y$ (V_c) or $\ell \to 1$ (V_r). Lastly, the negative contribution to $v = v(x,y)$ does not affect the model validity, as it produces the further constraint:

$$x \;\le\; \widetilde{x_{_M}}(y) \;=\; \left[\frac{y\sqrt{1+y^2}}{(\sqrt{1+y^2}-1)^2}\right]^{\frac{1}{3}} \tag{S8}$$

fulfilling $x \le \inf(x_{_M}, \widetilde{x_{_M}}) = x_{_M}(y)$.

2.2 Three-Descriptor Analysis of Bipyramid Shapes

Our geometric inversion model, as extensively detailed in the main text, is based on three key approximate assumptions. We have demonstrated that these simplifications significantly streamline the model without meaningful repercussions on the predictability of computational simulations (see Figure S4). They are: 1.) the model is projective and two-

dimensional; 2.) pentagonal symmetry is neglected, a circular base is used instead; 3.) lateral curvature radii are disregarded by juxtaposing two biconical structures. For the sake of generality, in this supplementary information, we introduce a less restrictive analysis in 3D, which may prove useful for future developments. To prevent confusion with the 2D model, the symbolic notation that follows is (and should be) kept distinct from that employed in the main text.

Consider thus the scheme in Figure S2, illustrating a pentagonal base of side length $\overline{\mathsf{L}}$, afterwards rounded at its vertices with equal junctions of radius ρ.

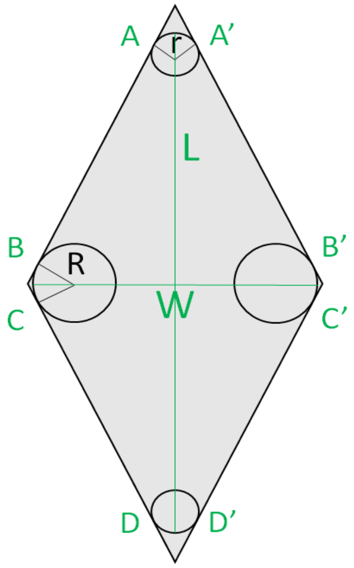

Figure S2: Longitudinal projection scheme of a bipyramid both rounded at the tips (radii of curvature r) and lateral sides (R). The real nanoparticle profile is depicted by $ABCDD'C'B'A'$, with given experimental values of width (W) and length (L). $\overline{W}$ and $\overline{L}$ here specify the maximum dimensions (tip-to-tip, side-to-side) belonging to the reference rhombus.

As in the inversion 2D model, the radii delimiting circle arcs are chosen to fall perpendicularly to the pentagon side. Let $\overline{W}$ be the maximum width, i.e. the distance between two non-nearest neighbour vertices, then:

$$\overline{\mathsf{L}} = \frac{1}{2}\sec\frac{\pi}{5}\,\overline{W}\,, \quad \overline{\mathsf{R}} = \frac{1}{2}\csc\frac{2\pi}{5}\,\overline{W}\,, \quad \overline{\mathsf{A}} = \frac{1}{4}\csc\frac{\pi}{5}\,\overline{W} \qquad \text{(S9)}$$

$\overline{\mathsf{R}}$ and $\overline{\mathsf{A}}$ being respectively the distance of any vertex from the pentagon center, and the apothem. These distances at the base and along the biconical axis reduce upon rounding as:

$$\mathsf{L} \;=\; \overline{\mathsf{L}} \;-\; \tfrac{2\pi}{5}\,\tau\rho\;, \quad \mathsf{R} \;=\; \overline{\mathsf{R}} \;-\; \sigma\rho\;, \quad \mathsf{A} \;=\; \overline{\mathsf{A}} \tag{S10}$$

with:

$$\tau \;=\; 5\tan\tfrac{\pi}{5} - \pi \;\approx\; 0.4911\;, \quad \sigma \;=\; \sec\tfrac{\pi}{5} - 1 \;\approx\; 0.2361\;, \tag{S11}$$

perimeter and area changing accordingly as:

$$\mathsf{P} \;=\; \overline{\mathsf{P}} \;-\; 2\tau\rho \tag{S12}$$

$$\mathcal{A} \;=\; \overline{\mathcal{A}} \;-\; \tau\rho^2 \tag{S13}$$

with unrounded values:

$$\overline{\mathsf{P}} \;=\; \tfrac{5}{2}\,\overline{W}\sec\tfrac{\pi}{5}\;, \quad \overline{\mathcal{A}} \;=\; \tfrac{5}{8}\,\overline{W}^2\csc\tfrac{2\pi}{5}\;. \tag{S14}$$

Figure S2 reports instead a scheme for the longitudinal bipyramid projection, either rounded on the tipped or lateral side with radii r and R, respectively.

It is inscribed into a rhombus, with smaller diagonal $\overline{W}$, the larger diagonal $\overline{L}$ denoting the maximum inter-tip distance. The rhombus aspect ratio of the perfect bipyramid:

$$\overline{\rho} \;=\; \frac{\overline{L}}{\overline{W}} \tag{S15}$$

is fixing the tip and side angles, α and β, as:

$$\cot\tfrac{\alpha}{2} \;=\; \tan\tfrac{\beta}{2} \;=\; \overline{\rho}\;, \tag{S16}$$

Some trigonometric algebra returns the longitudinal perimeter and surface area, i.e.:

$$\tfrac{1}{2}P \;=\; \tfrac{1}{2}\overline{P} \;-\; r\,\mathsf{T}(\overline{\rho}) \;-\; R\,\mathsf{T}(\overline{\rho}^{\,-1}) \;-\; r\,\overline{\rho} \;-\; R\,\overline{\rho}^{\,-1} \tag{S17}$$

$$A \;=\; \overline{A} \;-\; 2r^2\,\mathsf{T}(\overline{\rho}) \;-\; 2R^2\,\mathsf{T}(\overline{\rho}^{\,-1}) \tag{S18}$$

with $\mathsf{T}(x) \equiv x - \arctan x$ and, obviously:

$$\tfrac{1}{4}\overline{P}^2 \;=\; \overline{W}^2 + \overline{L}^2 \;=\; 4\overline{D}^2\;, \quad \overline{A} \;=\; \tfrac{1}{2}\overline{W}\,\overline{L} \tag{S19}$$

$\overline{D}$ being the rhombus side length. The shape transition here is characterized by three descriptors, two longitudinally symmetric with respect to each other:

$$\mathsf{S}_r \;\equiv\; 1 \;-\; \frac{2\lambda}{\overline{L}}\;, \quad \mathsf{S}_R \;\equiv\; 1 \;-\; \frac{2\Lambda}{\overline{W}} \tag{S20}$$

and one transverse:

$$\mathsf{S}_\rho \;\equiv\; \frac{\mathsf{R} - \overline{\mathsf{A}}}{\overline{\mathsf{R}} - \overline{\mathsf{A}}} \tag{S21}$$

In the first case, $\lambda = \lambda(r)$ and $\Lambda = \Lambda(R)$ represent the (minimum) distances from the rounding to the (nearest) tips, obeying:

$$\frac{\lambda(r)}{2r}\,\frac{\overline{W}}{\overline{D}} \;=\; \frac{\Lambda(r)}{2R}\,\frac{\overline{L}}{\overline{D}} \;=\; 1\;, \tag{S22}$$

thus implying ($\varrho = r, R$):

$$\mathsf{S}_\varrho \;=\; 1 \;-\; \frac{2\varrho}{\overline{L}}\sqrt{1+\overline{\rho}^{\,2}} \;=\; 1 \;-\; \frac{\overline{P}}{2\overline{A}}\,\varrho \tag{S23}$$

One immediately notices that the last equation is correct, as it returns for $\mathsf{S}_{\overline{\varrho}} = 0$ the formula for the radius of the circumference inscribed to any regular polygon, $\overline{\varrho} = 2\overline{A}/\overline{P}$, in terms of

which the two shape descriptors may be simply expressed as:

$$\mathsf{S}_r \;\equiv\; 1 \;-\; \frac{r}{\overline{\varrho}}\,, \quad \mathsf{S}_R \;\equiv\; 1 \;-\; \frac{R}{\overline{\varrho}} \tag{S24}$$

They can be also rearranged in terms of circularity, $C = 4\pi A/P^2$, experimental width (W) and length (L), i.e.:

$$\frac{\mathsf{S}_r}{\sqrt{1+\overline{\rho}^2}} \;=\; \frac{2r}{\overline{L}} \;=\; \left(1 - \frac{L}{\overline{L}}\right)\frac{1}{\sqrt{1+\overline{\rho}^2}-1} \tag{S25}$$

$$\frac{\mathsf{S}_R}{\sqrt{1+\overline{\rho}^{\,-2}}} \;=\; \frac{2R}{\overline{W}} \;=\; \left(1 - \frac{W}{\overline{W}}\right)\frac{1}{\sqrt{1+\overline{\rho}^{\,-2}}-1} \tag{S26}$$

or, in terms of circularity ($\overline{C}$):

$$\left(\frac{2\pi r}{\overline{P}}\right)\frac{1}{\mathsf{S}_r} \;=\; \left(\frac{2\pi R}{\overline{P}}\right)\frac{1}{\mathsf{S}_R} \;\equiv\; \overline{C} \;=\; 4\pi\frac{\overline{A}}{\overline{P}^2} \tag{S27}$$

The transverse descriptor instead obeys more simply:

$$\mathsf{S}_\rho \;=\; 1 \;-\; \frac{\sigma\rho}{\overline{\mathsf{R}}-\overline{\mathsf{A}}} \;=\; 1 \;-\; \frac{\rho}{\overline{\mathsf{A}}} \tag{S28}$$

as it has to be, since the equation $\mathsf{S}_\rho = 1$:

$$\rho \;\leq\; \overline{\mathsf{A}} \tag{S29}$$

represents the circumference inscribed to the pentagon base. The other two specular bounds, $S_r = 1$ and $S_R = 1$, return:

$$r \;\leq\; \frac{\overline{L}/2}{\sqrt{1+\overline{\rho}^2}}\;\;,\;\; R \;\leq\; \frac{\overline{W}/2}{\sqrt{1+\overline{\rho}^{\,-2}}} \tag{S30}$$

in agreement with the following limits:

$$\lim_{\overline{W} \vee \overline{L} \to 0^+} r \; = \; 0^+, \quad \lim_{\overline{W} \to +\infty} r \; = \; \tfrac{\overline{L}}{2}\,, \quad \lim_{\overline{L} \to +\infty} r \; = \; \tfrac{\overline{W}}{2}\,, \quad \lim_{\overline{W} \wedge \overline{L} \to d > 0} r \; = \; \tfrac{d}{2\sqrt{2}} \tag{S31}$$

and identically in R. The last limiting behaviour gives, still consistently, the radius of a circumference inscribed to a square of diagonal d.

2.3 Model Aspect Ratios

In our projective model, two aspect ratios are considered. The first (ℓ_n) represents the actual aspect ratio of the nanoparticle, while the second (y) is a conjectural parameter that enables the embedding of the nanoparticle within a biconical structure. For this reason, the latter has been termed cusp-to-cusp aspect ratio, referring to the distance between the two bicone vertices (L^*).

To compare the two concepts, let us examine their ratio, as predicted by the model:

$$\frac{\ell_n}{y} \; = \; 1 \; + \; \frac{x}{y}\left(1 - \sqrt{y^2+1}\right) \tag{S32}$$

which is nothing other than the real particle length compared to L^*, i.e. $L_n/L^* < 1$. When the second quantity on the right-hand side is small compared to one, i.e.:

$$x \; \ll \; \frac{y}{|1 - \sqrt{y^2+1}|} \; = \; 1 \; + \; y^{-1} \; + \; \tfrac{1}{2}y^{-2} \; - \; \tfrac{1}{8}y^{-4} \; + \; O(6) \tag{S33}$$

the two aspect ratios (or lengths) are approaching each other. Otherwise we may observe a significant deviation from unity, as reported in Figure S3 for the two parameterizations of Eq. (S32) in y and x.

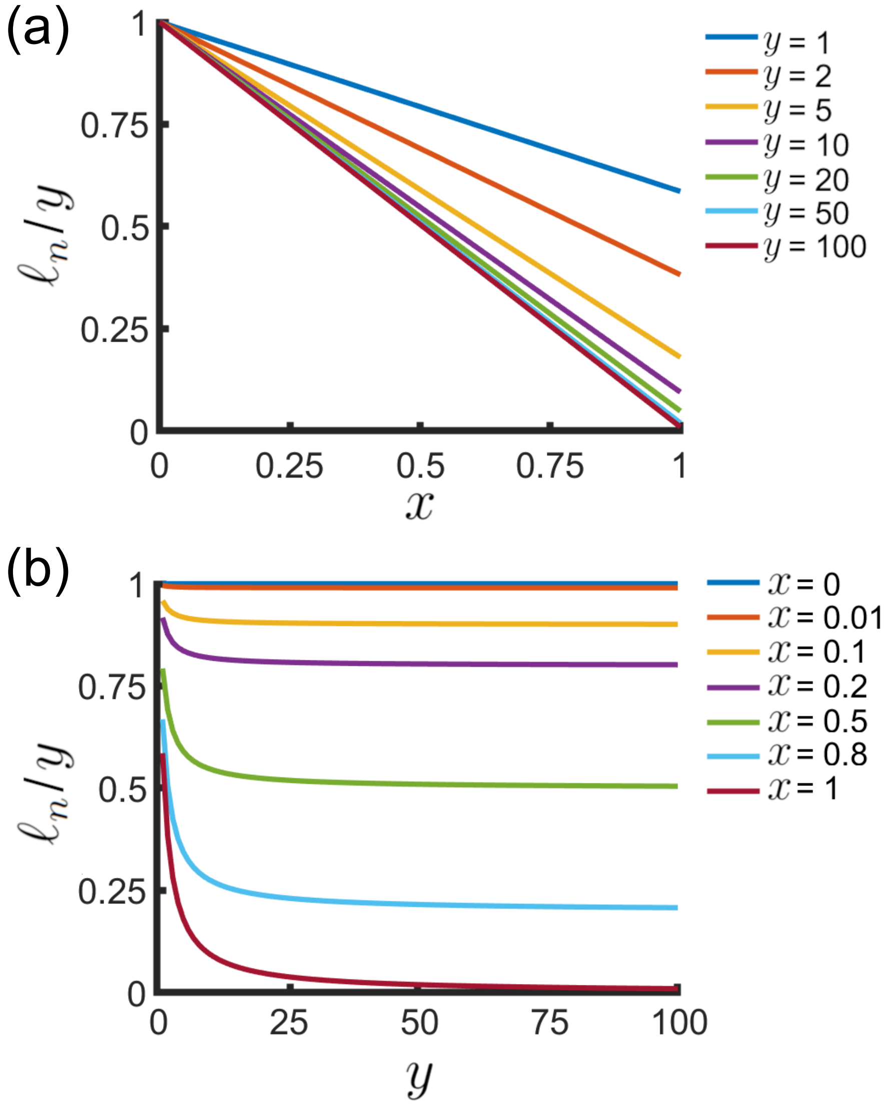

Figure S3: Parametric representation of Eq. (S32). Ratio between nanoparticle and cusp-to-cusp aspect ratios ℓ_n/y (a) as a function of x (parameterized by y) and (b) y (parameterized by x). The two quantities only coincide in the limit of $x \to 0^+$ (zero curvature radius).

3 C. Simulation of Optical Properties

Numerical simulations of the electromagnetic response of 3D bipyramid models, rebuilt by the geometric inversion method in 2D, are performed with the boundary element method[4] as implemented in the MNPBEM toolbox.[5] Particle surfaces are discretized in a number of surface elements (typically around 10^3-10^4) that ensures convergence of the computed electromagnetic quantities. Current and charge distributions over the surface are determined by solving the integral equations that result from imposing proper boundary conditions to Maxwell's equations. The electromagnetic field and the optical quantities of interest then can be computed accordingly.

In order to simulate a random particle orientation with respect to the incoming beam during measurements, calculations are performed by averaging over different polarizations of the incident light excitation. Optical constants of Au are taken from the established literature on this subject.[6] The surrounding medium is assumed to be water ($n_m = 1.333$) to mimic the as-synthesized Au bipyramids in a colloidal aqueous solution.

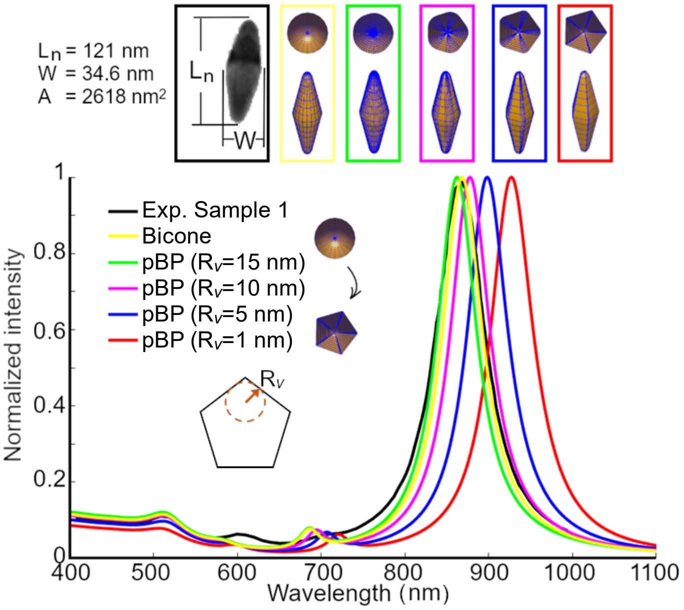

Figure S4: Comparative plot of the normalized experimental absorbance spectrum of Sample 1 (black) and numerical extinction spectra of pentagonal bipyramid particles (pBP) with variation of the radius of the pentagonal-base vertices (R_v) from 1 nm to 15 nm (red, blue, magenta, and green), and the biconical particle (yellow), for fixed values of the net length (L_n), width (W), and projected area (A) of the AuBP. Insets show the TEM image of a Sample-1 AuPB and the 3D particle models.

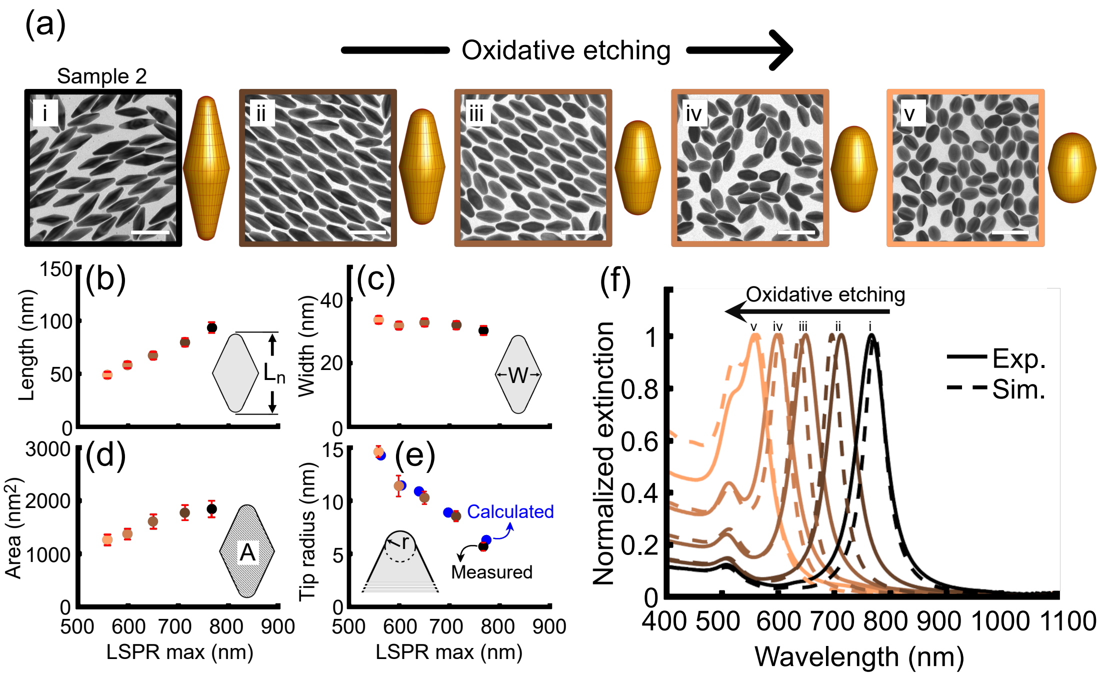

Figure S5: Oxidative etching of AuBPs, samples of Series 2. (a) TEM micrographs (scale bars = 100 nm) and the corresponding 3D models. Increasing degrees of oxidation are indicated by roman numerals from i to v, with i representing no etching. (b) Length, (c) width, (d) projected area, and (e) tip radius determined by analysing TEM micrographs. The blue circles in (e) represent the calculated tip radius obtained by implementing the geometric inversion method with the knowledge of length, width, and area data. (f) Experimental absorbance spectra (solid lines) along the computed extinction cross sections of the rebuilt 3D objects.

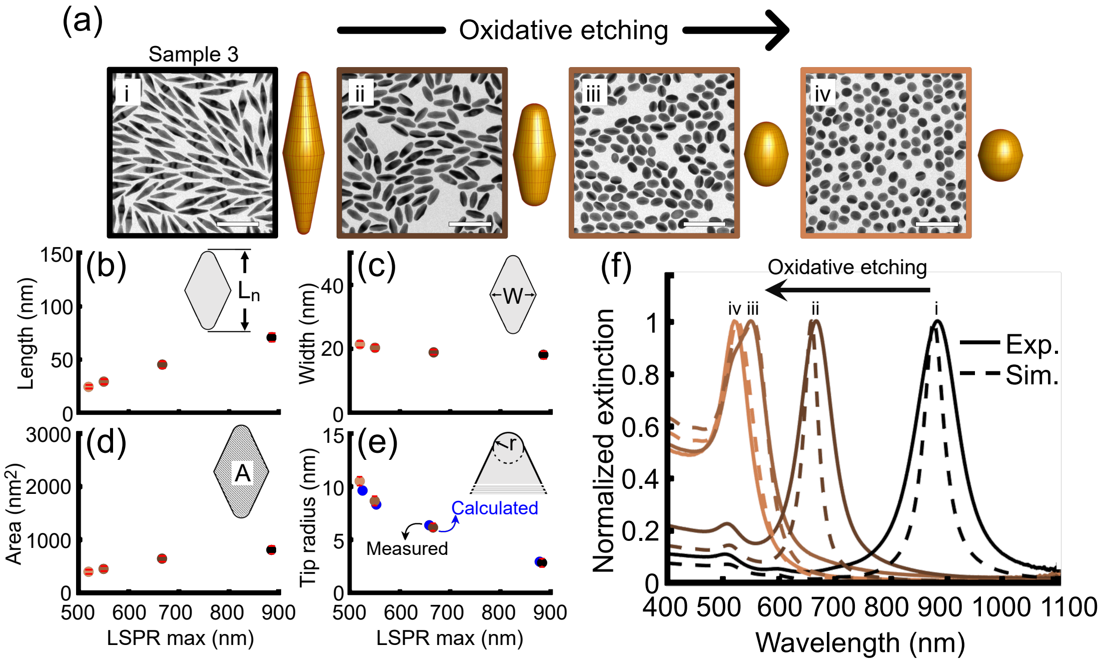

Figure S6: Oxidative etching of AuBPs, samples of Series 3. (a) TEM micrographs (scale bars = 100 nm) and the corresponding 3D models. Increasing degrees of oxidation are indicated by roman numerals from i to iv, with i representing no etching. (b) Length, (c) width, (d) projected area, and (e) tip radius determined by analysing TEM micrographs. The blue circles in (e) represent the calculated tip radius obtained by implementing the geometric inversion method with the knowledge of length, width, and area data. (f) Experimental absorbance spectra (solid lines) along the computed extinction cross sections spectra of the rebuilt 3D objects.

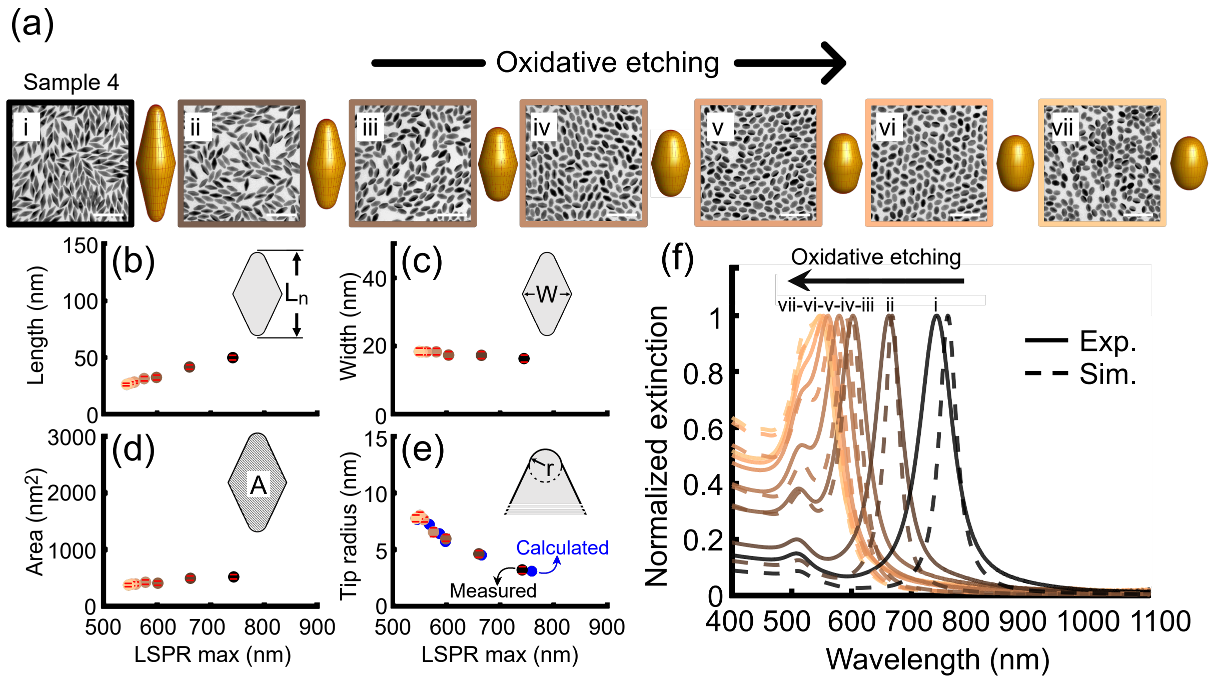

Figure S7: Oxidative etching of AuBPs, samples of Series 4. (a) TEM micrographs (scale bars = 100 nm) and the corresponding 3D models. Increasing degrees of oxidation are indicated by roman numerals from i to vii, with i representing no etching. (b) Length, (c) width, (d) projected area, and (e) tip radius determined by analysing TEM micrographs. The blue circles in (e) represent the calculated tip radius obtained by implementing the geometric inversion method with the knowledge of length, width, and area data. (f) Experimental absorbance spectra (solid lines) along the computed extinction cross sections of the rebuilt 3D objects.

Table S2: Average net length ($\overline{L}_n$), average width ($\overline{W}$), average projected area ($\overline{A}$), calculated tip radius (r), calculated cusp-to-cusp length (L^*), and shape descriptors x and y in the oxidative etching of samples of Series 1 (1-i to 1-vi), 2 (2-i to 2-v), 3 (3-i to 3-iv), and 4 (4-i to 4-vii).

Sample	$\overline{L}_n$ [nm]	$\overline{W}$ [nm]	$\overline{A}$ [nm^2]	r [nm]	L^* [nm]	x	y
1-i	121.0	34.6	2615.7	5.5	162.9	0.32	4.71
1-ii	120.0	35.3	2669.6	5.9	164.4	0.33	4.66
1-iii	112.4	35.1	2583.9	7.0	165.9	0.40	4.73
1-iv	98.4	34.9	2386.0	8.9	168.4	0.51	4.83
1-v	90.6	34.0	2239.5	10.1	177.0	0.59	5.20
1-vi	70.9	35.2	1904.3	12.8	174.6	0.73	4.95
2-i	92.7	30.0	1843.7	6.3	140.3	0.42	4.67
2-ii	79.0	31.7	1773.7	8.9	143.7	0.56	4.54
2-iii	66.7	32.6	1607.0	10.9	142.4	0.67	4.37
2-iv	57.8	31.6	1373.7	11.4	136.0	0.72	4.31
2-v	48.6	33.4	1265.2	14.2	157.2	0.85	4.71
3-i	70.9	18.2	818.2	3.0	97.5	0.33	5.34
3-ii	45.4	19.0	652.0	6.4	103.1	0.67	5.41
3-iii	29.8	20.4	465.3	8.3	82.1	0.82	4.02
3-iv	24.9	21.5	410.2	9.7	79.0	0.90	3.67
4-i	49.0	16.0	504.0	3.0	69.7	0.37	4.36
4-ii	41.0	17.0	480.0	4.4	68.7	0.52	4.04
4-iii	32.0	17.0	395.0	5.6	64.5	0.65	3.80
4-iv	31.0	18.0	410.0	6.3	66.3	0.70	3.68
4-v	28.0	18.0	383.0	7.1	72.4	0.78	4.02
4-vi	27.0	18.0	381.0	7.7	88.6	0.85	4.92
4-vii	26.0	18.0	361.0	7.5	76.5	0.83	4.25

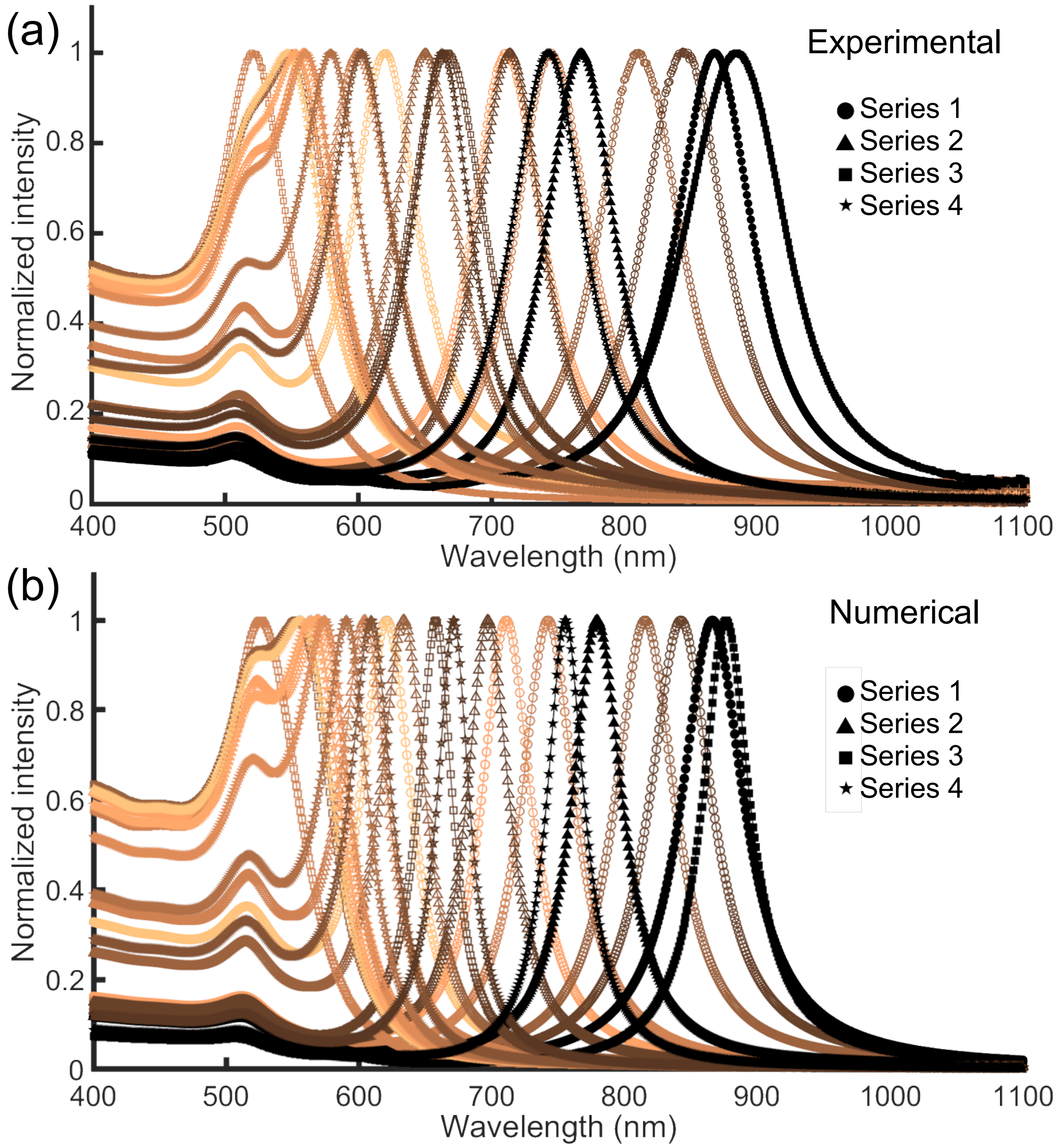

Figure S8: (a) Normalized experimental absorbance spectra and (b) numerical extinction spectra of oxidative-etched Series. Circle, triangle, square and star markers represent respectively the samples for Series 1, 2, 3, and 4. Spectra in black denote samples before oxidative etching, while spectra transitioning from dark to light copper colors depict increasing levels of oxidative etching.

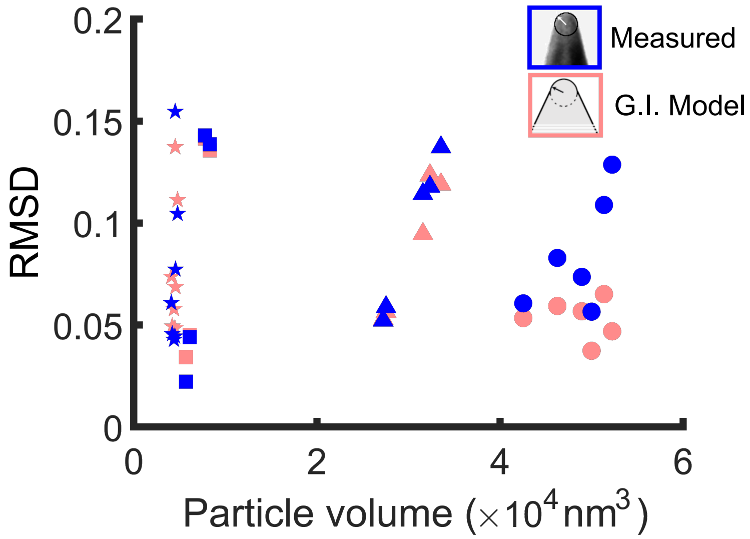

Figure S9: The root-mean-square deviation (RMSD) of simulated extinction spectra relative to experimental results is shown for the samples of the four oxidation series against the particle volume calculated by Eq. 6 in main text ($V = W^3 v$). The tip radius is determined: a.) from TEM micrographs (blue markers) and b.) by the geometric inversion model (light-red markers). Note that there is a higher deviation in the former measurement method (a). The deviation is calculated using the formula: RMSD $= \sqrt{(\sum_i (I_{i,exp} - I_{i,sim})^2)/N}$, where the sum runs over $N = 701$ wavelength samples in the spectral range, and $(I_{i,exp} - I_{i,sim})^2$ is the squared difference between experimental and calculated normalized intensities at each wavelength sample.

References

(1) Sánchez-Iglesias, A.; Winckelmans, N.; Altantzis, T.; Bals, S.; Grzelczak, M.; Liz-Marzán, L. M. High-Yield Seeded Growth of Monodisperse Pentatwinned Gold Nanoparticles through Thermally Induced Seed Twinning. *J. Am. Chem. Soc.* **2017**, *139*, 107–110.

(2) Rodríguez-Fernández, J.; Pérez-Juste, J.; Mulvaney, P.; Liz-Marzán, L. M. Spatially-directed Oxidation of Gold Nanoparticles by Au(III)-CTAB Complexes. *J. Phys. Chem. B* **2005**, *109*, 14257–14261.

(3) Zhou, W.; Lim, Y.; Lin, H.; Lee, S.; Li, Y.; Huang, Z.; Du, J. S.; Lee, B.; Wang, S.; Sánchez-Iglesias, A.; Grzelczak, M.; Liz-Marzán, L. M.; Glotzer, S. C.; Mirkin, C. A. Colloidal Quasicrystals Engineered with DNA. 2024.

(4) De Abajo, F. G.; Howie, A. Retarded Field Calculation of Electron Energy Loss in Inhomogeneous Dielectrics. *Phys. Rev. B* **2002**, *65*, 115418.

(5) Hohenester, U.; Trügler, A. MNPBEM–A Matlab Toolbox for the Simulation of Plasmonic nanoparticles. *Comput. Phys. Commun.* **2012**, *183*, 370–381.

(6) Johnson, P. B.; Christy, R. W. Optical Constants of the Noble Metals. *Phys. Rev. B* **1972**, *6*, 4370–4379.
